# Silver Nanoparticles as a Tool for the Study of Spontaneous Aggregation of Immunoglobulin Monoclonal Free Light Chains

**DOI:** 10.3390/ijms22189703

**Published:** 2021-09-08

**Authors:** Anna Lizoń, Joanna Tisończyk, Marta Gajewska, Ryszard Drożdż

**Affiliations:** 1 Department of Medical Diagnostics, Faculty of Pharmacy, Jagiellonian University Medical College, Medyczna 9, 30-688 Cracow, Poland; joanna.tisonczyk@uj.edu.pl; 2 Academic Centre for Materials and Nanotechnology, AGH University of Science and Technology, Aleja Adama Mickiewicza 30, 30-059 Crakow, Poland; marta.gajewska@agh.edu.pl

**Keywords:** nanotechnology, protein misfolding, nanoparticle–protein interactions, amyloidosis

## Abstract

Some misfolded proteins, e.g., immunoglobulin monoclonal free light chains (FLC), tend to form fibrils. Protein deposits in tissue may lead to amyloidosis and dysfunction of different organs. There is currently no technique allowing for the identification of FLC that are prone to aggregate. The development of such a method would enable the early selection of patients at high risk of developing amyloidosis. The aim of this study was to investigate whether silver nanoparticles (AgNPs) could be a useful tool to study the process of aggregation of FLC and their susceptibility to form the protein deposits. Mixtures of AgNPs and urine samples from patients with multiple myeloma were prepared. To evaluate the aggregation process of nanoparticles coated with proteins, UV-visible spectroscopy, transmission electron microscopy, and the original laser light scattering method were used. It has been shown that some clones of FLC spontaneously triggered aggregation of the nanoparticles, while in the presence of others, the nanoparticle solution became hyperstable. This is probably due to the structure of the chains themselves, unique protein-AgNPs interactions and perhaps correlates with the tendency of some FLC clones to form deposits. Nanoparticle technology has proven to be helpful in identifying clones of immunoglobulin FLC that tend to aggregate.

## 1. Introduction

Immunoglobulin monoclonal free light chains (FLC) are involved in several protein deposition diseases, e.g., light chain amyloidosis (AL amyloidosis) and light chain deposition disease. In AL amyloidosis, FLC form characteristic amyloid fibrils which are deposited in tissues, while in light chain deposition disease, protein deposits are amorphous [1]. Amyloidosis is a group of protein conformational diseases caused by misfolding, aggregation and deposition of different proteins, mainly in the extracellular spaces of organs and tissues. These protein deposits have characteristic form of insoluble, non-branching, rigid fibrils [2]. Amyloid fibrils have a diameter of 7–13 nm and similar core structure consisting of, mainly, anti-parallel β-strands which form sheets [3]. These fibrils are characterized by their capacity to bind the dye Congo red and show apple-green birefringence under polarized light [4]. There have been more then 30 different proteins identified so far as a possible cause of amyloidosis, and, with the use of mass spectrometry-based proteomic analysis, the number of amyloid precursors is still increasing [2].

The most common form of acquired systemic amyloidosis is AL amyloidosis. About 70% of patients with amyloidosis suffer from AL amyloidosis. It is characterized by a deposition of misfolded monoclonal light chains (kappa or lambda) that are secreted from a malignant plasma cell clone. Some monoclonal free light chains do not conform an alfa-helical configuration typical for most proteins, but rather form β-pleated sheets. As a result of changed protein conformation, amyloid fibrils are formed and deposited in tissues [5]. Different organs can be affected by amyloid deposits. The leading cause of morbidity and mortality in amyloidosis is cardiac involvement, which has the greatest effect on survival [3]. The mechanism of tissue damage in amyloidosis is not fully understood. There are several theories, e.g., the replacement of intercellular substance by amyloid deposits, atrophy and degeneration of cells induced by amyloid fibrils or the toxicity of non-fibrillar states of amyloidogenic proteins [6]. In spite of recent advances in AL amyloidosis diagnostic tools and treatment, the early mortality rate is still high. Almost 30% of all AL amyloidosis patients die within a few months of diagnosis. It also happens that even if an appropriate diagnosis is made and treatment is initiated, the disease is often very advanced [7].

The molecular mechanisms that lead to the formation of amyloid are not well understood. It is not fully known why some proteins form amyloid while others do not. The ability to form deposits and, possibly, the tissue tropism are related to the structure of the protein. As a natural process, each patient produces immunoglobulin light chains that have undergone antigen-driven selection. The great variety of protein aggregates can be a result of both unique primary amino acids sequence and different environmental factors [8].

It is known that patients with AL amyloidosis may have the monoclonal free light chains presented in their sera years before the clinical manifestation of the disease [9,10]. Initially, patients with some forms of plasma cell dyscrasia, i.e., monoclonal gammopathy of unknown significance (MGUS), are not treated, despite the fact that some of them will develop full clinical manifestations of multiple myeloma including protein deposition diseases. It is important to confirm the presence of amyloid precursor protein as early as possible. There have been some works on the technique that could help to identify the tendency of protein to form amyloid aggregates. A good diagnostic tool has still not been found [10]. The development of a method that could identify “amyloid-prone” protein would allow for the early selection of patients at higher risk of disease and the implementation of effective treatment. With the introduction of new drugs, the survival rate of amyloidosis patients has increased significantly.

Noble metal nanoparticles, due to their unique optical, chemical and photothermal properties, are increasingly used in medicine [11]. Nanoparticles interact with different biomolecules, especially proteins, in a specific way, depending on the structure and physicochemical characteristics of both nanoparticles and proteins. This provides the ability to use nanoparticles to study the interactions between proteins [12]. These interactions are extremely important, particularly in the case of the development of severe diseases associated with the formation of protein deposits in the tissues (amyloidosis). AgNPs interact with proteins and, as a result of this interaction, the function and structure of proteins can be changed. This fact is currently a matter of study because nanoparticles are more and more frequently introduced into human bodies for biomedical applications [13]. Changes in protein functions can be harmful for organisms [14]. However, on the other hand, the change of structure and/or function of protein can also have therapeutic or diagnostic applications. Described changes in the structure of proteins upon coating of nanoparticles were associated with, among others, breaking disulfide bonds, reducing the amount of alpha helices and increasing the number of beta sheets within protein molecules [15,16]. It is well known that this type of conformational change is responsible for protein deposit diseases (amyloidosis) [17]. The identification of proteins that tend to change conformation can help in the diagnosis of serious life-threatening diseases. There are studies showing that nanoparticles can be a factor that initiates protein aggregation. Due to their unique optical properties, AgNPs also enable the direct observation of the initial stage of protein aggregation [18,19].

The aim of this study was to investigate whether silver nanoparticles can be a useful tool for identifying FLC clones that tend to interact and aggregate. This model can possibly be used in patients with multiple myeloma who are at high risk of developing amyloidosis.

## 2. Results

### 2.1. Characteristics of Silver Nanoparticles

**Figure 1:**
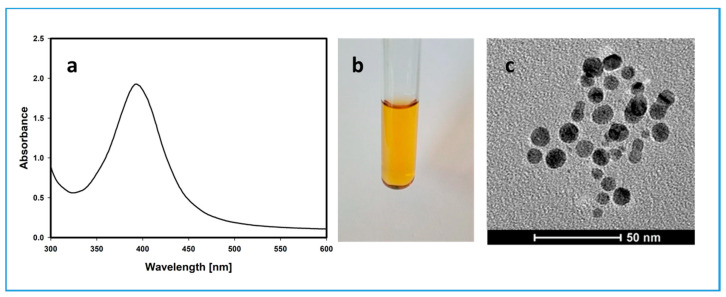
Silver nanoparticles prepared by a chemical reduction of silver ions using borohydride; (**a**) characteristic UV-visible (UV/Vis) spectrum, with the maximum absorbance at 395 nm; (**b**) color of the solution; (**c**) transmission electron microscope image of the synthesized silver nanoparticles.

The characteristics of the synthesized citrate-stabilized silver nanoparticles was performed using transmission electron microscopy (TEM) and UV-visible (UV/Vis) spectrophotometry (Figure 1).

The maximum of the localized surface plasmon resonance (LSPR) spectrum was 395 nm and the average diameter of AgNPs, based on TEM, was 9.62 nm (median 9.2 nm, SD 3.4 nm) [12,20]. To estimate the molar concentration of the nanoparticle solution, LSPR maximum and the molar extinction coefficient of the synthesized AgNPs (5.56 × 10^8^ M^−1^ cm^−1^) were taken into account [21]. Assessed molar concentration of the solution was about 10 nM.

### 2.2. The Use of Silver Nanoparticles to Study the Process of Interaction of Free Monoclonal Light Chains of Immunoglobulins

As immunoglobulin monoclonal light chains are thermodynamically unstable and some clones of FLC tend to spontaneously aggregate [22], a model was developed to investigate the interaction between immunoglobulin free light chains coated on silver nanoparticles.

**Figure 2:**
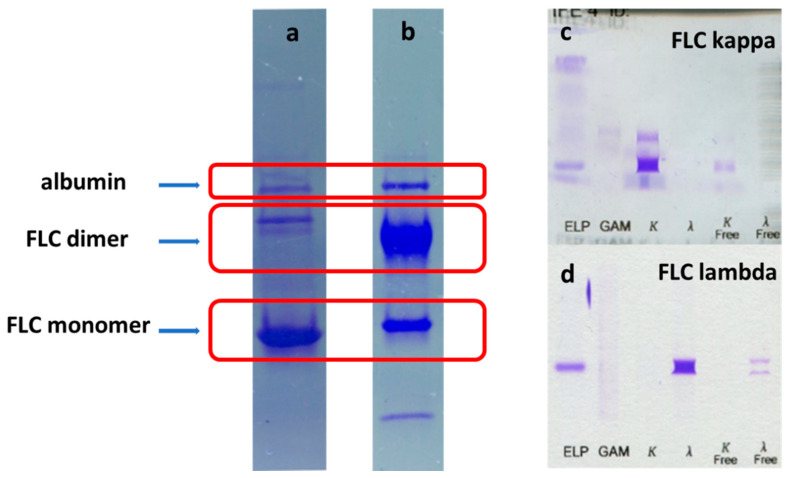
Representative examples of electrophoretic patterns of urine samples used in the study (**a**,**b**). As seen, the main component of urine samples were monomers or dimers of the immunoglobulin light chains. They were proved to be kappa (**c**) or lambda (**d**) free light chains—based on the immunofixation.

For the functionalization of AgNPs, selected urine samples from patients with multiple myeloma were used. The main urine protein component were monomers or dimers of immunoglobulin free light chains (Figure 2).

Materials from 37 patients were used in this study, including 16 samples with FLC type lambda and 21 with kappa-type FLC. FLC lambda concentrations in the urine samples were in the range of 54.8–5370 mg/L while FLC kappa concentrations were between 100–3900 mg/L. Measurements of FLC concentrations were made with the commercial Freelite assay from Binding Site.

For each clone, six samples were prepared to obtain the final FLC concentrations in the samples: 0 mg/L (0 nM), 0.2 mg/L (10 nM), 1 mg/L (50 nM), 3 mg/L (150 nM), 5 mg/L (250 nM) and 10 mg/L (500 nM). The process of monoclonal light chains aggregation was investigated by UV/Vis spectroscopy, TEM and direct laser light scattering.

**Figure 3:**
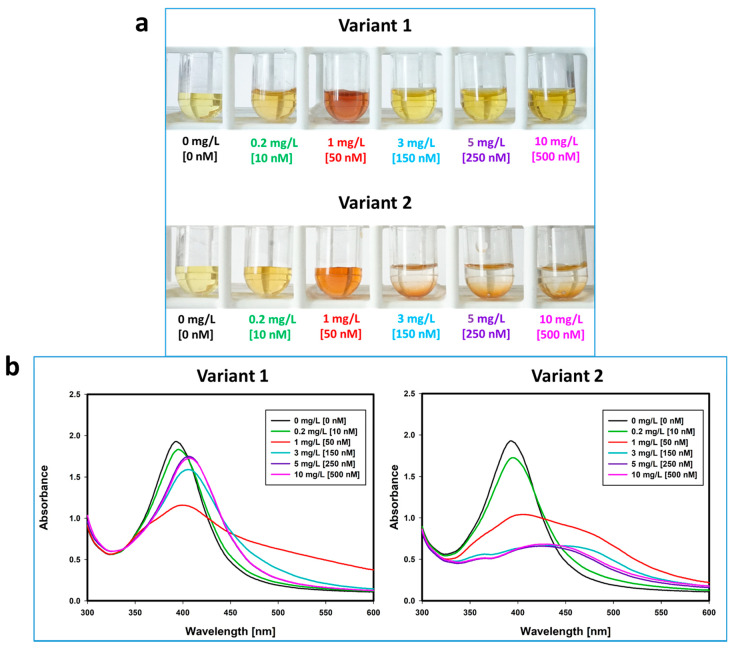
Macroscopic changes (**a**) and localized surface plasmon resonance spectra changes (**b**) observed as a result of functionalizing of silver nanoparticles (10 nM) with various clones of free monoclonal immunoglobulin light chains. In both variants, at the free light chains concentration of 1 mg/L (50 nM), a characteristic red color of the solution was observed, and similar (variant 1 and 2) UV/Vis spectrum changes occurred: a decrease in the absorbance maximum, a broadening of the spectrum and a red-shift. At higher concentrations of the free light chains (above 3 mg/L (150 nM)) in variant 1 the solution was stable and yellow. There was a slight decrease in the maximum absorbance (in relation to the citrate-stabilized nanoparticles) and its shift to the right (red-shift), without broadening of the spectrum. In variant 2, at higher concentrations of the free light chains (above 3 mg/L (150 nM)), aggregation of nanostructures occurred and the spectral line was flattened.

When analyzing the reactions that occur in solutions of silver nanoparticles functionalized with monoclonal free light chains, two characteristic patterns of changes were observed. In both variants, the solution changed color from yellow to red at the FLC concentration of 1 mg/L (50 nM) (Figure 3a). At this FLC concentration (1 mg/mL), specific LSPR changes took place resulting in a red color of solution. In the UV/Vis spectrum, there was a characteristic decrease in the maximum absorbance, its red-shift and widening of the AgNPs plasmon resonance spectrum (Figure 3b). At higher concentrations of FLC (3 mg/L (150 nM), 5 mg/L (250 nM), 10 mg/L (500 nM)), the nanoparticle solution was either stabilized—a protein corona was formed around the nanoparticles and the characteristic effect of LSPR change was blocked (variant 1)—or spontaneously aggregated (variant 2).

In variant 1, with increasing concentrations of FLC (above 3 mg/L (150 nM)), there were spectral changes suggesting the formation of a protein corona around the nanoparticles; a slight decrease in absorbance maximum (in relation to citrate-stabilized nanoparticles) and a red-shift, without spectral broadening (Figure 3b). The solutions were yellow. Analyzing the molar concentrations of the reagents, the number of protein molecules per one nanoparticle in these solutions were 15:1, 25:1 and 50:1. In variant 2, the absorbance spectra curves flattened at high FLC concentrations and there was a macroscopically visible aggregation of nanostructures.

The presented patterns of changes occurred without relation to the type of free chains. Variant 1 was observed in 12/16 FLC lambda samples and in 18/21 FLC kappa samples. The second pattern related to nanoparticle aggregation occurred in 4/16 FLC lambda samples and in 3/21 FLC kappa samples.

To sum up, as a result of coating silver nanoparticles with immunoglobulin free light chains in a molar ratio of 5:1 (FLC:AgNPs), the LSPR and the color of the solution changed (from yellow to red). At higher ratios of FLC to AgNPs (15:1, 25:1, 50:1), in some clones, a stable protein corona was formed around the nanoparticles, while other clones interacted and caused aggregation of nanostructures. Aggregation of the nanoparticles in this method is the result of interactions between proteins attached to nanoparticles. In vivo, the proteins that form amyloid interact and aggregate. It is not possible to observe the aggregation of the protein in solution used in the study, but indirectly—as a visible effect—the aggregation of nanoparticles is the result of protein aggregation.

**Figure 4:**
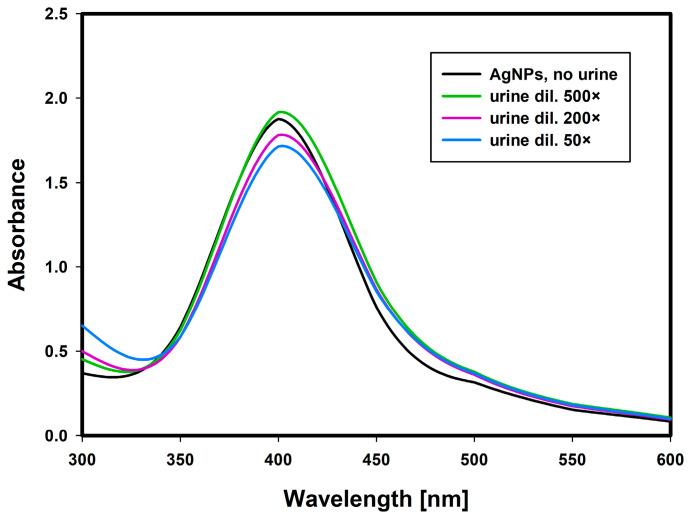
UV/Vis spectra of silver nanoparticle solutions after addition of different volumes of urine samples from healthy donors—control study, representative example. The dilutions of urine samples were: 50×, 200×, 500× —the same as in the test with FLC-containing samples.

To confirm that the ionic strength resulting from the addition of various volumes of urine to the nanoparticles solution did not affect the results of the study and that only FLC generate nanoparticle aggregation, control experiments were performed with urine samples from healthy donors. The urine protein profiles of healthy donors were confirmed by SDS-electrophoresis. Figure 4 shows UV/Vis spectra of AgNPs solutions after addition of different volumes of urine samples (dilutions 50, 200 and 500 ×—the same as in the test FLC-containing samples). There were no significant differences in the absorbance spectra of the control samples and no nanoparticle aggregation occurred in any of them, which indicates that the aggregation process in the test samples was due to the presence and interaction of specific FLC clones.

**Figure 5:**
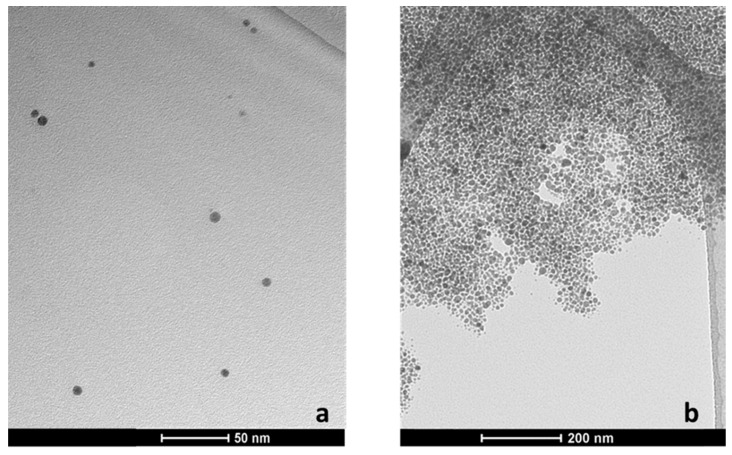
Transmission electron microscope images of silver nanoparticles solutions; (**a**) silver nanoparticles coated with “non-aggregating” free light chains (10 mg/L (500 nM)), stable, with a protein corona (variant 1); (**b**) nanoparticle aggregates—silver nanoparticles coated with aggregation-inducing free light chains (10 mg/L (500 nM)) (variant 2).

The interaction of free monoclonal light chains coated on silver nanoparticles was also investigated using TEM imaging (Figure 5). Figure 5a shows single silver nanoparticles coated with immunoglobulin free light chains (10 mg/L (500 nM)), stable, with a protein corona (which corresponds to variant 1 described above). Figure 5b shows aggregates of nanoparticles formed as a result of interactions between immunoglobulin light chains (variant 2).

**Figure 6:**
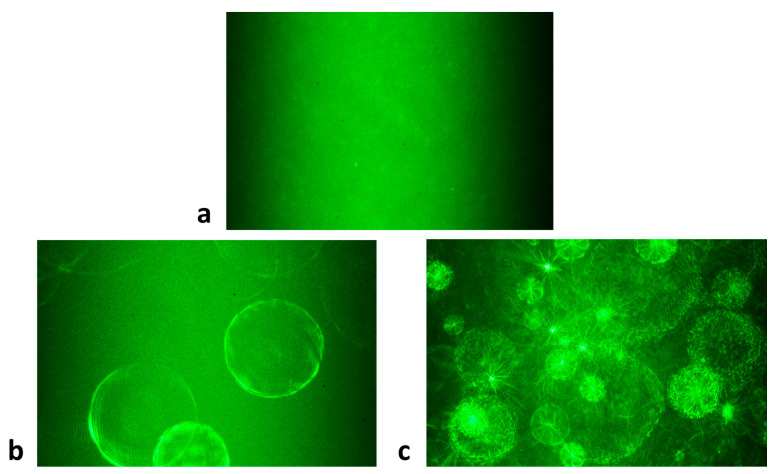
Scattering patterns of monochromatic green semiconductor laser light, passing through: (**a**) citrate-stabilized silver nanoparticles; (**b**) silver nanoparticles covered with a large protein corona (variant 1); (**c**) nanoparticle aggregates (variant 2).

Assessing the interactions between immunoglobulin free light chains, the patterns of laser light scattering by the resulting mixtures were also analyzed. A narrow beam of laser light passing through the solution of citrate-stabilized silver nanoparticles gave a homogenous scattering pattern (Figure 6a). When other solutions were investigated, it turned out that the protein corona (variant 1) formed in the presence of high concentrations of FLC (150 nM, 250 nM, 500 nM) gave the image of characteristic concentric circles (Figure 6b). The aggregates resulting from the variant 2 gave intense light scattering patterns (like “flashes of fireworks”) (Figure 6c).

**Figure 7:**
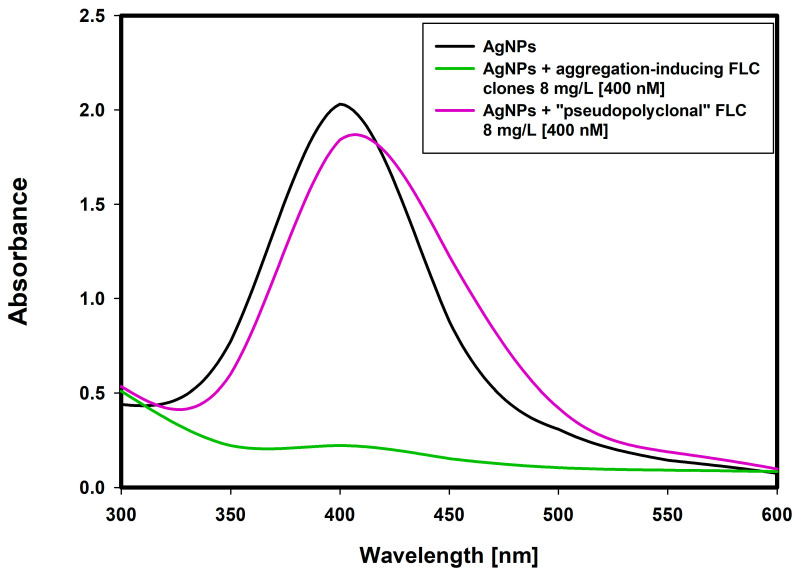
UV/Vis spectrum changes that occur after coating silver nanoparticles with free light chains (8 mg/L, (400 nM)): aggregation-inducing free light chains clone (green line) and “pseudopolyclonal” free light chains (violet line). The “pseudopolyclonal” free light chains mixture does not cause aggregation of nanoparticles.

Aggregation of immunoglobulin monoclonal FLC is related to their clonality. There are no reports in the literature of the formation of amyloid structures in patients with polyclonal hypergammaglobulinemia. When AgNPs were functionalized with a mixture of four aggregation-inducing clones of FLC, the aggregation process was not observed, which confirmed that aggregation is related to interaction of monoclonal particles with defined structure (Figure 7). Long amyloid fibril is made of repeating structures of a homogeneous protein with precisely defined conformation enabling the formation of bonds parallel to the axis of the fiber [17]. The presence of four different types of proteins with different structures disrupts the process of interaction and aggregation.

## 3. Discussion

Immunoglobulin monoclonal free light chains are a very diverse group of proteins. They are characterized by enormous (related to the antigen-binding function) amino acid variability and wide pI range. In a study by M. Diemert et al. [23], the pI values of immunoglobulin free light chains from 92 patients with Bence-Jones proteinuria were analyzed. The results ranged from 3 to 9, of which 55% of patients had pI values of <6, and 45% of >6. Such a wide distribution of pI suggests that different FLC clones may behave differently. Free light chains are low molecular weight incomplete fragments of immunoglobulins which, under physiological conditions, due to rapid glomerular filtration, are present in the blood at concentrations of 10 mg/L. In physiological conditions, the free light chains present in the blood (produced in some excess to the heavy chains) are polyclonal proteins that “do not match” and do not tend to aggregate. In some patients, in the course of monoclonal gammopathies, a homogeneous fraction of monoclonal protein tends to aggregate and accumulate in the tissues. Monoclonal free light chains of immunoglobulins, sometimes conformationally altered as a result of proteolysis or other factors, are thermodynamically unstable and tend to spontaneously form extracellular aggregates [24,25,26]. Some forms of this pathological protein (FLC) present in myeloma patients are responsible for the protein deposits disease—amyloidosis [27]. The mechanism of amyloid formation is related to the disturbance of protein folding. Due to some factors, the native, thermodynamically unstable form of the protein adopts a β-sheet structure, from which amyloid fibers are then formed. These deposits can be visualized by electron microscopy and polarized microscopy after staining with Congo red, which is used in diagnostics [28].

Currently, the most common form of amyloidosis is AL amyloidosis [2]. It occurs in approximately 10–15% of multiple myeloma cases but, as there are currently no good screening tools for amyloidosis, it seems that the number of correct diagnoses is underestimated. AL amyloidosis may also result from a benign primary proliferation of plasmocytes, meeting the criteria for the diagnosis of monoclonal gammopathy of undetermined significance. The clinical symptoms of amyloidosis depend on the organ localization of the deposits. As a consequence, the deposition of pathological proteins leads to impairment of the functions of the affected organs. In the case of AL amyloidosis, the organs most commonly involved are the heart, kidneys, liver and tongue. The molecular mechanism of this damage is not fully understood; its explanation based solely on the replacement of intercellular substance by amyloid deposits is insufficient. There are theories that it is not the fibers themselves that exhibit cytotoxic properties, but amyloid fiber precursors. In some patients, amyloidogenic monoclonal free immunoglobulin light chains bind to factor X of the coagulation system, leading to ecchymosis [29].

Excessive synthesis (several dozen grams per day in some patients) of light chains is not synonymous with a predisposition to amyloid formation. It is not fully known what causes the deposition of pathological proteins in a particular patient. The amino acid sequence of the variable part of the chain certainly plays an important role. Certain amino acid sequences common to many types of light chain amyloid precursors have been identified [30,31]. Beta sheet structure fragments are characteristic for amyloid fiber precursors [10]. However, taking into account the fact that recreating the process of the formation of protein deposits in vitro can be very difficult, the conclusion is that many environmental factors also contribute to the process of amyloidogenesis. Only the interaction between the protein and the environment results in the formation of fibrils or amorphous deposits [32]. Attention is drawn to the importance of the concentration of pathological deposition precursors [33]. The environmental pH plays an important role in the process of creating protein deposits [34]. There is a relationship between the isoelectric point of proteins and the type of deposits. In the case of AL amyloidosis, the isoelectric point of the chains is within the limits 3.8–5.2, although there are also exceptions to this rule [35].

We now know that most cases of AL amyloidosis are diagnosed too late. When amyloid deposits form in the tissue of the heart muscle, most patients die within a few months. AL amyloidosis can be effectively treated, and early diagnosis is a prerequisite for successful treatment. Currently, there are no laboratory tests to assess the susceptibility of free light chains to aggregation and amyloid fibril formation [10]. The development of a test that could assess the ability of specific clones of free light chains to aggregate in patients with monoclonal gammopathy would be of great diagnostic importance in the treatment of this disease.

The aim of this study was to investigate whether silver nanoparticles can be a useful tool to study the process of aggregation of free light chains and their susceptibility to form the protein deposits. Selected urine samples from patients with multiple myeloma were used to coat the citrate-stabilized silver nanoparticles. Due to interactions between proteins, in some cases nanoparticles aggregated. This model can possibly help to identify proteins that tend to aggregate.

As the results of the work, it turned out that the effects of coating citrate-stabilized silver nanoparticles with free light chains were different. Indeed, they depended on the clone of monoclonal free light chains and the amount of FLC that was used. Some clones caused the nanoparticles’ aggregation, while others caused the hyperstablility of the AgNPs in solution. As a result of coating silver nanoparticles with free light chains in a molar ratio of 5:1 (FLC:AgNPs), the LSPR and the color of the solution changed (from yellow to red). At higher ratios of FLC to AgNPs (15:1, 25:1, 50:1), in some clones, a stable protein corona was formed around the nanoparticles, while the remaining ones interacted and caused aggregation of nanostructures. The element of the right protein–nanoparticle ratio turned out to be extremely important. The aggregation of monoclonal free light chains coated on silver nanoparticles was accompanied by changes in the LSPR spectrum. The aggregation process was also confirmed by the TEM method and by analyzing the scattering patterns of laser light.

Noble metal nanoparticles are very efficient at light scattering. Different imaging techniques have been used to observe the light scattering: dark-field microscopy, conventional confocal microscopy. These methods are very sensitive and make it possible to observe single nanoparticles in live cells [36,37]. The dark-field microscopy equipped with a tungsten lamp was extensively used to directly analyze the true color images of the scattered light of a single gold nanoparticle. Color analysis of the scattered light was performed by decoding RGB (red, green, blue) color space of the digital images [38]. Silver nanoparticles scatter about 10 times more strongly than gold. Additionally, it has been shown that monochromatic laser-band light illumination lowers the detection limit by about 10 times than that with white light illumination [39].

The method used in this study—with the monochromatic laser light—gave different patterns of light scattering corresponding to different nanoparticle–protein interactions. This unique method using monochromatic laser light may be considered as another type of methodology associated with laser light scattering; the obtained patterns were unique and related to this specific methodology. The analysis of light scattering patterns is an inexpensive, relatively simple research method and can be used for the qualitative study of the process of interaction between proteins in real time [12].

It is possible that, when bound to silver nanoparticles, free light chains undergo conformational changes favoring the structure of the beta sheet (such a phenomenon has been described for other proteins [15,18]). Silver nanoparticles are thus potentially the catalyst for the first slowest stage of amyloid formation. A similar effect was described by Gilan S et al. [40]. They observed that zero-valent iron nanoparticles with an appropriate dose could shorten the lag phase of α-synuclein fibrillation and increase the rate of fibrillation.

Changes in the structure and function of proteins resulting from their coating on nanoparticles have been repeatedly described in the literature.

The study of Ban D and Paul S [41] showed that as a result of the interaction of starch-stabilized AgNPs with bovine alpha-lactalbumin (BLA, 14.2 kD), the secondary structure of the protein was changed and its biological activity was lost. The BLA in such a conjugate (AgNPs-BLA) contained fewer exposed nonpolar residues on the surface and thus the hydrophobicity of the protein surface was reduced. BLA molecules became resistant to proteinase K. This observation proves that the introduction of nanoparticles into a biological system can make a protein highly resistant to proteinase K, which can prevent its normal degradation process and thus lead to the accumulation of non-functional proteins in cells. Vertegel et al. studied changes in the structure and activity of lysozyme after coating silicon nanoparticles with it [42]. Depending on the size of nanoparticles, the effect of lysozyme–SiNPs interactions was a reduction in the number of alpha-helices in the protein structure. The activity of the enzyme coated on the nanoparticles was lower than that of free protein, in proportion to the lost alpha helices. Similar changes in the protein structure—a decrease in the amount of alpha helices and an increase in the amount of beta sheets—were observed by Li Shang et al. [16], studying the interaction of albumin with gold nanoparticles. The intensity of the changes depended on the pH of the environment; at higher pH, the changes were more intense.

The above examples illustrate the complexity of nanoparticle–protein interactions, taking into account the diversity of the protein structure. The effects of nanoparticle–protein interactions are different depending on the structure of the protein, size, concentration, and method of nanoparticle stabilization [43].

This hypothesis is confirmed by the work of Zhang et al., who showed that gold nanoparticles can induce conformational changes and aggregation of lysozyme at physiological pH [15]. Changes in the structure were associated with, among others, breaking disulfide bonds, reducing the number of alpha helices and increasing the number of beta sheets within the lysozyme molecule. It also turned out that the coating of gold nanoparticles with thiolated polyethylene glycol (PEG) in an appropriate concentration inhibited the process of protein aggregation. This confirms how important the method of nanoparticle stabilization is for the process of functionalization and interactions with proteins. Another peptide used in studies of amyloid formation is insulin. The formation of amyloid-like structures is found in some patients with diabetes type 2. In their work, Sukhanova et al. showed that CdSe/ZnS quantum dots of a certain size (12 nm) stabilized with PEG-OH (negatively charged, zeta potential −6 mV) induced changes in the insulin secondary structure and, consequently, the formation of amyloid fibers [24].

It has been also shown that it is nanoparticles that can induce conformational changes leading to the formation of fibrils [19,44,45]. For example, Linse et al. showed that nanoparticles (copolymer particles, cerium oxide particles, quantum dots, and carbon nanotubes) enhance the probability of the appearance of a critical nucleus for nucleation of protein fibrils from human β2-microglobulin 2-microglobulin [19].

Therefore, it cannot be ruled out that conformational changes related to FLC adsorption on the surface of silver nanoparticles accelerate the aggregation process and amyloid formation. This simple model (FLC-coated nanoparticles) can potentially serve as an assay for selecting free light chain clones that tend to spontaneously aggregate. Importantly, AgNPs prepared according to the Creighton method were stable for several months, the functionalization procedure was simple, and the aggregation process was also visible by direct observation.

Aggregation of immunoglobulin monoclonal free light chains is related to their clonality. Transverse (to the axis of the amyloid fiber) aggregation of free monoclonal light chains is possible when exposed fragments with a beta sheet structure form a clone-specific interlocking complementary structure. There are no reports in the literature of the formation of amyloid structures in patients with polyclonal hypergammaglobulinemia. Protein molecules from different clones have distinct, mismatched beta-sheet binding fragments. In order to verify the above hypothesis, the selected aggregation-inducing FLC clones were mixed and added to AgNPs solution. It turned out that the “pseudopolyclonal” mixture of free light chains did not cause aggregation of nanoparticles. This confirms the utility of the nanoparticle system in identifying clones of free immunoglobulin light chains that tend to agglutinate and possibly form amyloid.

To sum up, nanoparticle technology has proven to be helpful in identifying clones of monoclonal free light chains that tend to aggregate. This may be related to the susceptibility of some FLC clones to the formation of amyloid deposits.

## 4. Materials and Methods

### 4.1. Materials

Urine samples from 37 patients with multiple myeloma were used in this study, including 16 samples with FLC lambda and 21 with FLC kappa type. As a control group urines samples from 40 healthy blood donors were collected.

AgNO_3_, NaBH_4_ and trisodium citrate were purchased from Sigma-Aldrich (St. Louis, MO, USA).

SDS electrophoresis was performed using Sigma-Aldrich TruPAGE Precast Gels 4–20%, TruPAGE SDS Sample Buffer and TruPAGE TEA-Tricine SDS Running Buffer. Color Prestained Protein Standard (broad range 11–245 kDa) was from New England BioLabs (Ipswich, MA, USA). Separations were performed using Mini-PROTEAN MP (300V) Bio-Rad (Hercules, CA, USA). Gels were stained using Brilliant Blue G Stain (Sigma-Aldrich).

Urine protein immunofixation was performed on an automatic Interlab G26 (Rome, Italy) electrophoretic platform using dedicated kit (SRE626K) and antibodies targeting the heavy chains of human immunoglobulins (IgG, IgM, and IgA), and their light chains, kappa and lambda, both bound and free—all the regents from Interlab (Rome, Italy).

### 4.2. Synthesis of Silver Nanoparticles 

The synthesis of silver nanoparticles was based on the method described by Creighton [46]. This method is based on the reduction of silver ions using sodium borohydride. Silver nanoparticles were additionally stabilized with sodium citrate. The procedure was as follows: to 19 mL of chilled distilled water, 0.5 mL of an aqueous solution of AgNO_3_ (0.01 M) and 0.5 mL of trisodium citrate (0.01 M) were added. Then, 0.6 mL of sodium borohydride (0.01 M) solution was injected to the above solution drop by drop while stirring vigorously. The synthesis temperature was 0 °C. After synthesis, the vigorous magnetic stirring was maintained for 3 min. As a result of synthesis, a stable, light yellow AgNPs solution was obtained. The solution was stored at 4 °C until use.

### 4.3. Characterization of Urine Samples

Characterization of urine samples was performed by SDS electrophoresis using commercially available 4–20% gradient gels from Sigma. Urine protein immunofixation—to determine the type of free light chains—was carried out on commercial immunofixation gels from InterLab using anti-FLC kappa and anti-FLC lambda antibodies (Lambda/Kappa Bound and Free antiserum and Lambda/Kappa Free antiserum by Interlab). Measurements of FLC concentrations were made with the commercial Freelite assay from Binding Site.

### 4.4. Functionalization of AgNPs with FLC

In order to functionalize silver nanoparticles with FLC, different urine volumes were added to the AgNPs solution. Six samples were prepared for each patient; the urine volume was added to 1 mL of silver nanoparticles to obtain the desired final concentrations of FLC in the samples: 0 mg/L (0 nM), 0.2 mg/L (10 nM), 1 mg/L (50 nM), 3 mg/L (150 nM), 5 mg/L (250 nM) and 10 mg/L (500 nM). All samples were incubated at 37 °C overnight.

### 4.5. UV/Vis Spectroscopy

Citrate-stabilized silver nanoparticles and FLC-functionalized silver nanoparticles were analyzed using UV/Vis spectroscopy (Multiscan Sky, Thermo Scientific, Waltham, MA, USA). The wavelength range used in the study was between 350–600 nm. Pathlength adjustment to 1 cm was performed.

### 4.6. Transmission Electron Microscopy

Citrate-stabilized nanoparticles, FLC-coated nanoparticles and aggregates (size and shape) were imaged using Tecnai TF20 X-TWIN (200 kV)—a high-resolution transmission electron microscope equipped with field emission gun (FEG) (Thermo Scientific, Waltham, MA, USA). TEM sample preparation consisted of drop-casting and drying a nanoparticle suspension on carbon-coated copper grids.

### 4.7. Laser Light Scattering 

All the solutions (citrate-stabilized AgNPs, FLC-coated AgNPs, nanoparticle aggregates) were analyzed with the use of a self-made measurement system for the observation of the laser light scattering patterns. The system included a horizontally placed optical microscope and a long working distance objective lens placed in the revolver (40×). Analyzed nanoparticle solutions were inserted into standard plastic cuvettes (1 cm optical path). A green semiconductor laser (532 nm) was located above the sample. A Sony alpha 6000 camera (Minato, Tokio, Japan) was used to record the light scattering patterns.

## 5. Conclusions

The results of the study show that immunoglobulin monoclonal free light chains are a very heterogeneous group of proteins. Different clones of FLC behave differently. This probably depends on the structure of the chains themselves and perhaps correlates with the tendency to form deposits. It is possible that FLC adsorption on silver nanoparticles is accompanied by conformational changes stimulating the formation of or revealing the β-sheet structure. They therefore act as a catalyst in the first slowest stage of amyloid formation.

The technology using silver nanoparticles, in selecting the appropriate imaging techniques, can be a useful method, offering new possibilities for tracking reactions related to protein aggregation, e.g., in the study of amyloidosis and other diseases related to the formation of protein deposits.

## Data Availability

Not applicable.
